# Molecular Virologic and Clinical Characteristics of a Chikungunya Fever Outbreak in La Romana, Dominican Republic, 2014

**DOI:** 10.1371/journal.pntd.0005189

**Published:** 2016-12-28

**Authors:** Rose M. Langsjoen, Rebecca J. Rubinstein, Tiffany F. Kautz, Albert J. Auguste, Jesse H. Erasmus, Liddy Kiaty-Figueroa, Renessa Gerhardt, David Lin, Kumar L. Hari, Ravi Jain, Nicolas Ruiz, Antonio E. Muruato, Jael Silfa, Franklin Bido, Matthew Dacso, Scott C. Weaver

**Affiliations:** 1 Institute for Human Infections and Immunity and Center for Tropical Diseases, University of Texas Medical Branch, Galveston, TX, United States of America; 2 Institute for Translational Sciences, University of Texas Medical Branch, Galveston, TX, United States of America; 3 Center for Global Health Education, University of Texas Medical Branch, Galveston, TX, United States of America; 4 Department of Microbiology & Immunology, University of Texas, Galveston, TX, United States of America; 5 Department of Pathology, University of Texas Medical Branch, Galveston, TX, United States of America; 6 cBio Inc., Fremont, CA, United States of America; 7 Hospital Dr. Francisco Gonzalvo, La Romana, Dominican Republic; 8 Hospital el Buen Samaritano, La Romana, Dominican Republic; George Washington University School of Medicine and Health Sciences, UNITED STATES

## Abstract

Since emerging in Saint Martin in 2013, chikungunya virus (CHIKV), an alphavirus transmitted by the *Aedes aegypti* mosquito, has infected approximately two million individuals in the Americas, with over 500,000 reported cases in the Dominican Republic (DR). CHIKV-infected patients typically present with a febrile syndrome including polyarthritis/polyarthralgia, and a macropapular rash, similar to those infected with dengue and Zika viruses, and malaria. Nevertheless, many Dominican cases are unconfirmed due to the unavailability and high cost of laboratory testing and the absence of specific treatment for CHIKV infection. To obtain a more accurate representation of chikungunya fever (CHIKF) clinical signs and symptoms, and confirm the viral lineage responsible for the DR CHIKV outbreak, we tested 194 serum samples for CHIKV RNA and IgM antibodies from patients seen in a hospital in La Romana, DR using quantitative RT-PCR and IgM capture ELISA, and performed retrospective chart reviews. RNA and antibodies were detected in 49% and 24.7% of participants, respectively. Sequencing revealed that the CHIKV strain responsible for the La Romana outbreak belonged to the Asian/American lineage and grouped phylogenetically with recent Mexican and Trinidadian isolates. Our study shows that, while CHIKV-infected individuals were infrequently diagnosed with CHIKF, uninfected patients were never falsely diagnosed with CHIKF. Participants testing positive for CHIKV RNA were more likely to present with arthralgia, although it was reported in just 20.0% of CHIKF+ individuals. High percentages of respiratory (19.6%) signs and symptoms, especially among children, were noted, though it was not possible to determine whether individuals infected with CHIKV were co-infected with other pathogens. These results suggest that CHIKV may have been underdiagnosed during this outbreak, and that CHIKF should be included in differential diagnoses of diverse undifferentiated febrile syndromes in the Americas.

## Author Summary

Chikungunya virus (CHIKV) is known for its ability to cause explosive outbreaks of flu-like illness followed by severe joint pain and swelling, which can persist for months to several years. We tested patient serum of both suspected and unsuspected chikungunya fever (CHIKF) cases collected at an emergency clinic in La Romana, Dominican Republic for markers of current or recent CHIKV infection, and showed through next generation sequencing that the causative CHIKV strain is closely related to other isolates from the Americas. After matching clinical outcomes with diagnostic data, we found that relatively few CHIKF patients presented with the joint symptoms typically associated with CHIKV infection in the past, which likely contributed to a notable frequency of CHIKF misdiagnosis. Other deviations from Old World CHIKF outbreaks were also discovered, including shifts in affected age groups and in the frequency of respiratory signs and symptoms. These data are particularly important for improving future surveillance endeavors, as well as the diagnosis and treatment of CHIKF in the Americas.

## Introduction

Chikungunya virus (CHIKV), a mosquito-borne virus in the family *Togaviridae*, genus *Alphavirus*, was first isolated in 1952 from a patient on the Makonde plateau that is now part of Tanzania. It derives its name from the Makonde term *kungunyala*, meaning roughly, “that which bends up” [1]. Urban chikungunya fever (CHIKF) outbreaks were first described in the late 1950s in Asia, transmitted by the mosquito vector *Aedes aegypti*. In 2005, CHIKV emerged again from enzootic circulation in Africa and spread to the Indian Ocean Basin, India and other Asian countries and also made incursions into Italy and France, which reported local transmission for the first time in Europe [2–6]. This unprecedented spread was partly credited to the ability of the CHIKV strains within the new Indian Ocean lineage (IOL) to adapt through E1 and E2 envelope glycoprotein substitutions to a new vector species, *Ae*. *albopictus*, which itself spread from Asia to many parts of the world since 1985 [7–9]. Then, in 2013, a CHIKV strain belonging to the same lineage that had been circulating in Asia at least since the 1950s (Asian lineage) was introduced into the Americas, first detected on the Caribbean island of St. Martin before spreading through South and Central America, including Mexico [10, 11]. Local transmission was reported in the U.S. in Florida and Texas after the arrival of infected travelers [12, 13]. To date, this New World introduction has resulted in over 1.9 million suspected cases of CHIKF in North, Central, and South America as well as the Caribbean Islands [Pan American Health Organization (PAHO) data]. An independent introduction of an East, Central, and South Africa (ECSA)-derived enzootic CHIKV strain from Africa was also reported in Brazil in 2014 [14].

Strains from all CHIKV lineages can cause explosive outbreaks of CHIKF, which is characterized by a generalized febrile syndrome typically including high fever, maculopapular rash, muscle and joint aches, headache, and malaise, which progresses to severe polyarthritis/polyarthralgia. Although CHIKF is generally self-limiting and sometimes resolves within a month of onset, up to 30–70% of CHIKF patients experience chronic and/or recurring arthritis lasting months to years [4].

Polyarthritis and polyarthralgia are hallmarks of CHIKF, and can range between 80% and 97% of patients reporting these symptoms during past epidemics [2, 3, 15–18]. However, data from New World CHIKV infections remain limited with few reports examining in detail patient signs and symptoms. Some studies suggest that infection with New World/Asian CHIKV lineage strains results in arthritic outcomes similar to those observed in previous epidemics, but with only limited data available for long-term joint sequelae [19].

**Fig 1:**
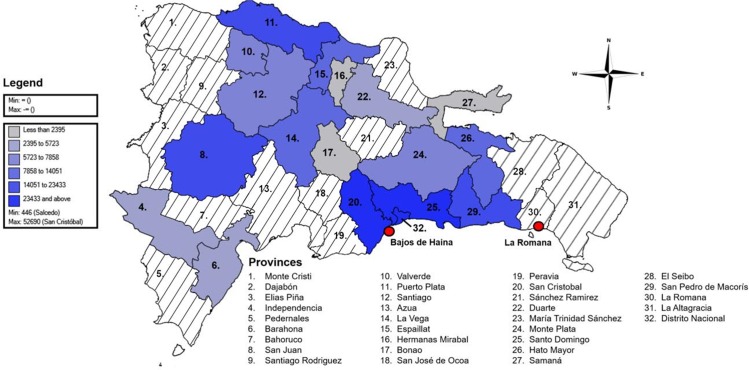
Distribution of total suspected cases of CHIKF reported in the Dominican Republic by province. A suspected case was defined by the presence of sudden fever and arthralgia. The color scheme classifies provinces by number of total suspected CHIKF cases, determined by summing the number of cases reported in MSP DIGEPI weekly bulletins and Chikungunya Outbreak Bulletins for each epidemiological weeks between February 16, 2014 and June 6, 2015. No exact case numbers were reported to MSP DIGEPI for provinces shaded in white. Number 32, Distrito Nacional, represents the national district, which does not pertain to a province. The city of La Romana and the port of Bajos de Haina, where the outbreak is suspected to have started, are highlighted in red. Map created using Epi Info^TM^ 7.1.5 software licensed by the Centers for Disease Control and Prevention (http://wwwn.cdc.gov/epiinfo/7/). MSP DIGEPI weekly bulletins publicly available through Minesterio de Salud Publica (http://digepisalud.gob.do/).

Among New World CHIKF outbreaks, the Dominican Republic has had the most suspected cases, with over 500,000 reported since 2014 [PAHO data] (Fig 1). Here, we describe an outbreak in the southeastern city of La Romana. In February, 2014, the Dominican Republic’s first suspected cases were reported in Nigua, a town in San Cristóbal Province, southwest of the capital. Suspected cases were defined by the Dominican Ministerio de Salud Pública (MSP) by acute febrile syndrome and polyarthralgia, though many patients also presented cervical, supraclavicular, and inguinal adenomegalies and facial, vulva and scrotal edema. It was hypothesized that CHIKV entered the country through Bajos de Haina, a port city located 2 km from Nigua. On April 3, 2014, the U.S. Centers for Disease Control and Prevention (CDC) confirmed CHIKV infection by detecting CHIKV-specific IgM antibodies in a patient’s blood [10]. Nationally, cases continued to rise, peaking between mid-July and mid-August with up to 45,000 new cases each week. After administering brief questionnaires in major cities, the MSP estimated attack rates ranging from 40% of the population-at-risk in Higüey to 81% in Azua de Compostela. In La Romana, up to 89% of households interviewed were suspected to have been affected by CHIKV as of August, 2014 [20]. Clinicians and patients reported a high fever and arthralgia in the wrists and ankles, the latter which lasted up to six months in middle-aged and elderly female patients. Nevertheless, little published information describes the rate of CHIKV seropositivity or CHIKF symptoms in the La Romana population.

Here, we report a case series of CHIKF patients in La Romana from June-August, 2014 during the CHIKF outbreak, following diagnostic tests of patient sera and retrospective chart reviews of signs and symptoms as well as clinical data associated with these patients, and genetic characterization of CHIKV isolates from patients.

## Methods

### Patient data and serum collection

Between June and August, 2014, serum collected for complete blood counts (CBC) was collected from discarded diagnostic blood samples of patients attended by the department of emergency medicine at Hospital el Buen Samaritano (HBS). The criteria for serum collection in HBS included patients for whom a complete blood count (CBC) was performed. A retrospective chart review was performed to collect patient data. Patient age, gender, symptoms, and CBC results were collected retrospectively wherever possible. For patients who were admitted into the hospital, a physical examination and medical history were recorded, including a history of the present illness and clinical and familial antecedents. All patient samples and data were assigned institution-specific identification numbers to ensure patient anonymity. Neither patient names nor clinic-assigned laboratory numbers were used, and patient age was used in lieu of birthdate, eliminating all potential identifying information from the study without compromising data integrity.

All patient data were deidentified and handled under University of Texas Medical Branch Institutional Review Board Protocol #15–0265.

### Quantitative RT-PCR

RNA was first isolated from serum samples using the ZR-96 Viral RNA Kit (Zymo Research, Orange, CA, USA) according to the manufacturer’s protocol. Quantitative reverse transcription PCR (RT-qPCR) was then performed using the TaqMan RNA-C_t_ 1-step Kit (Applied Biosystems, San Francisco, CA, USA) and previously described primers [21].

### CHIKV IgM ELISA

Serum samples were screened for anti-CHIKV IgM antibodies by enzyme-linked immunosorbent assay (ELISA) as previously described [22] using the CHIKjj Detect MAC-ELISA kit (InBios, Inc., Seattle, WA), which was validated by the CDC [23]. All samples were tested in duplicate and any inconclusive samples were retested.

### Plaque assay

African green monkey kidney cells (Vero; American Type Cell Culture, Bethesda, MD) were grown to 90–100% confluency in 12-well plates. Serum was diluted in Dulbecco's mimimal essential medium (DMEM) (supplemented with 5% FBS and from a series of 10-fold dilutions, medium was removed from plates, and 100uL of serum dilution was plated per well. After 60 minutes incubation, an overlay composed of DMEM and 0.2% agarose was added and incubated for 24–48 hours. Plates were then fixed with 10% formaldehyde for one hour before staining with crystal violet, and plaques counted.

### Sequencing and phylogenetic analysis

Ten serum samples obtained during the CHIKV RT-qPCRs with low RT-qPCR Ct values were submitted for Illumina HiSeq sequencing, without passaging, as previously described [19]. Viral genomes were assembled using the Abyss software [24]. Assembled contigs were checked using bowtie2 to align reads to the contigs followed by visualization using the integrative genomics viewer [25]. Genomic sequences were manually aligned with those representing all three genotypes downloaded from Genbank using Se-Al (http://tree.bio.ed.ac.uk/software/seal/), and non-coding sequences were removed from the alignment, resulting in a common length of 11,241 nt. The final data set comprised of 85 complete open reading frame sequences from 26 countries isolated during 1953–2014. A Bayesian maximum clade credibility (MCC) phylogeny was inferred using the GTR+G4 nucleotide substitution model with BEAST version 1.8.2 [26].

### Statistics

This study is primarily observational as it followed a series of CHIKF cases, and thus most data were reported in raw form. Contingency tables for admitted vs. outpatients were constructed with outpatients in the first row and hospitalized in the second, and those with symptoms/parameter in the first column and without in the second; relative risk analysis was then performed using Medcalc (MedCalc Software, Ostend, Belgium). Continuous variables were first tested for normalcy, normalized as needed, and then means were analyzed by either T-test or one-way ANOVA using SPSS software. Regression analyses were employed to ascertain correlation between C_T_ value and symptom frequency using SPSS software.

## Results

### Finalized sample pool

A total of 194 serum samples, collected between July and August, 2014, were included in the study; at the time of sample receipt, scientists were blinded to disease state of the patients sampled. Of those, 95 (49%) were positive for CHIKV RNA by RT-qPCR, 48 (24.7%) were positive for acute CHIKV antibodies by IgM ELISA, and 2 samples were positive for both (1%; Table 1). In total, 145/194 (74.7%) sera tested positive for recent CHIKV infection. C_T_ values for RNA-positive samples ranged between 34 and 17.39 (S1 Fig), and plaque assays on RNA-positive samples revealed infectious titers ranging between 2.9x10^5^ plaque forming units/mL to below the limit of detection of 1x10^2^ PFU/mL. Sequencing and phylogenetic analysis confirmed that the CHIKV isolates obtained from the DR were closely related to other isolates collected from Caribbean outbreaks, stemming from an Asian lineage strain (S2 Fig).

**Table 1:** Diagnostic outcomes for serum samples collected from patients in La Romana, Dominican Republic between July 2014 and August 2014

Parameter	n(percent)
Total samples	194
Samples CHIKV-positive	145(74.7)
by RT-qPCR	95(49.0)
by IgM ELISA	48(24.7)
By both RTq-PCR and IgM ELISA	2(1.0)

### Demographic data

**Fig 2:**
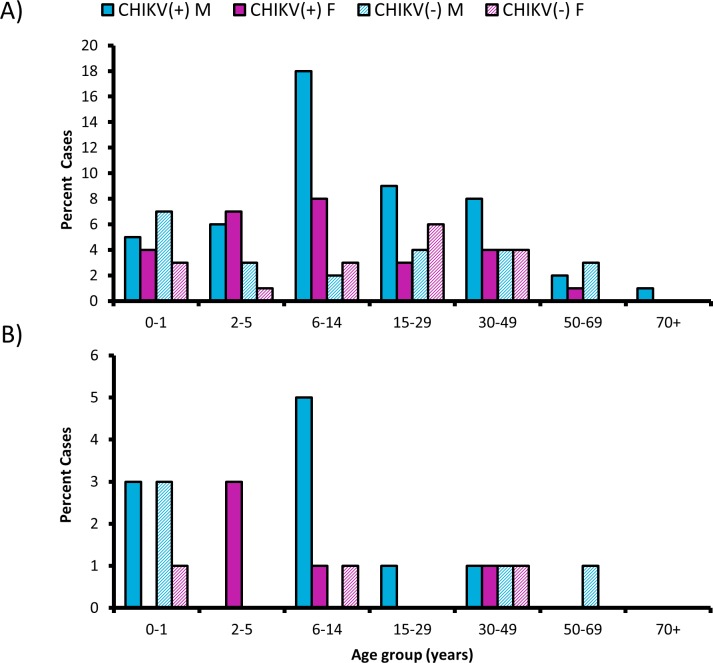
Age and gender distribution of patient sample pool. Serum from patients visiting an emergency clinic in the DR was tested for CHIKV RNA and IgM, and results were retrospectively matched to demographic data. A) The age and gender distribution of CHIKV-RNA positive [CHIKF(+)] and CHIKF(-) (both IgM and RNA negative) patients. The majority of patients were under the age of 50 for both CHIKF(+) and CHIKF(-) groups. B) The age and gender distribution of patients who were hospitalized.

After samples underwent diagnostic testing, de-identified patient data were analyzed for demographic characteristics such as gender, age, and hospital admission (Table 2). As inclusion criteria were not used to regulate sample submission, these data are strictly observational and are presented to describe the general clinical picture associated with this particular sample set. Only RNA-positive samples were included in these analyses because IgM can persist up to several months after infection, thus not necessarily indicating active infection. In total, demographic data were available for 75 CHIKF(+) (CHIKV RNA-positive) and 41 CHIKF(-) (both RNA- and IgM-negative) patients. More male samples were represented for both CHIKF(+) and CHIKF(-) patient groups. A broad age range of both CHIKF(+) and CHIKF(-) patients was represented, between 13 days and 85 years, with the majority of cases involving children between the ages of 6 and 14 years (Fig 2). The mean time from fever onset to clinic visit ranged from 2–7 days, with an average of 3.8 days for CHIKF(+) patients and 4.0 days for CHIKF(-) patients. Viremia was detectable up to 7 days post-onset of illness in CHIKF(+) patients. Patients in the CHIKF(-) group had a higher rate of hospitalization, with 18.7% of CHIKF(+) patients and 26.8% of CHIKF(-) patients requiring admission. The mean age and time of symptom onset (before visiting the clinic) were not significantly different between admitted and outpatient CHIKF(+) and CHIKF(-) groups (ANOVA with Tukey’s post-hoc, 0.27<p<0.93; Table A in S1 Tables), nor did gender significantly affect the relative risk (RR) of being hospitalized for either group.

**Table 2:** Demographic data for CHIKF(+) and CHIKF(-) patient serum samples collected from patients in La Romana, Dominican Republic July-August 2014, and associated relative risk.

Characteristic	CHIKV(+) value	CHIKV(-) value
**Male: Female (ratio)**	2	1.3
**Mean years of age, ± STD**	16.2**±**16.9	19.6**±**19.1
**Time from onset to hospital visit, mean days ± STD**	3.8**±**1.8	4**±**1.4
**Hospitalized n(%)**	15 (18.7)	8 (26.8)

### Symptoms, clinical diagnoses, and blood findings

In addition to demographic data, specific sign and symptom data were matched to samples (Table 3). These data were available for 48 CHIKF(+) patients and 19 CHIKF(-) patients. The average ages for patients with available symptom data were 16.5**±**16.4 years for CHIKF(+) patients and 29.6±23.3 years for CHIKF(-) patients. More symptom data were available for admitted patients, as hospital admission rates for patients with available symptom data were 31.2% for CHIKF(+) and 57.9% for CHIKF(-) patients; however, similar to demographic data, no demographic trait significantly affected the risk of hospitalization (Table B in S1 Tables). The most common feature of both CHIKF(+) and CHIKF(-) patients was fever (91.7% and 63.2%, respectively) averaging 39.4°C for CHIKF(+) patients, while the average fever for CHIKF(-) patients was slightly lower at 38.8°C. Complaints of arthralgia and myalgia were surprisingly low for CHIKF(+) patients at 20.0% and 13.3%, respectively. Despite the low frequency of these particular symptoms, they may still be considered of diagnostic value, as arthralgia and myalgia appeared to be CHIKV-specific, with no patients in the CHIKF(-) group exhibiting these symptoms. Several preexisting conditions were noted, the most prominent being pregnancy of greater than 12 weeks and hypertension However, because these occurred so infrequently, the effect of risk on hospital admission was not assessed.

**Table 3:** Signs and symptoms recorded for CHIKF-positive (and CHIKF-negative) patients for whom complete blood counts were ordered between July 2014 and August 2014 at Hospital Buen Samaritano, La Romana, Dominican Republic.

Symptom	CHIK(+)	CHIKV(-)
**TOTAL (no. patients)**	46	19
**Fever**	42 (91.7)	12 (63.2)
**Average (°C ± STD)**	39.4±0.5	38.8±0.3
Arthralgia*	6 (20)	0 (0)
Myalgia*	4 (13.3)	0 (0)
Headache*	7 (23.3)	5 (38.46)
**Enophthalmos**	5 (14.6)	1 (5)
**Malaise/fatigue**	4 (8.7)	2 (10.5)
**Rash**	0 (0)	0 (0)
**Dehydration**	14 (30.5)	5 (26.3)
**Gastrointestinal**^**A**^	7 (15.2)	7 (36.8)
**Respiratory**^**B**^	9 (19.6)	6 (31.6)
**PREXISTING CONDITIONS**		
**Pregnancy >12 weeks**^**†**^	2 (16.6)	1 (20.0)
**Hypertension**	1 (2.3)	1 (5.3)

*Patient data for children under 3 years of age not included

^†^Percent calculated from number of female patients of child-bearing age (9–45)

^A^Nausea, diarrhea, vomiting

^B^pneumonia, dyspnea, rhonchus, difficulty breathing, rhinorrhea

Values in n(percent) unless otherwise noted.

**Fig 3:**
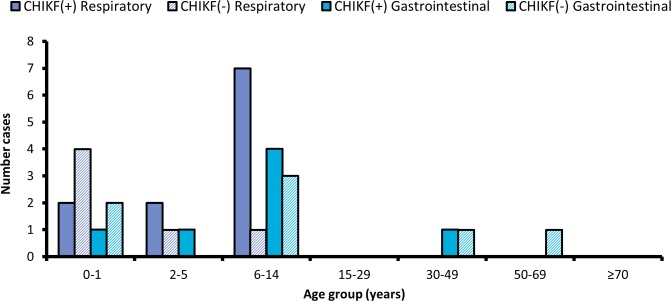
Distribution of respiratory and gastrointestinal symptoms by age. Serum from patients visiting an emergency clinic in the DR was tested for CHIKV RNA and IgM, and results were retrospectively matched to clinical features documented by physicians. Children and young adults below the age of 15 were the primary demographic groups presenting with respiratory and gastrointestinal symptoms for both CHIKF(+) (RNA-positive) and CHIKF(-) (both IgM and RNA negative) patients.

Other signs and symptoms less commonly associated with CHIKV infection can be broadly categorized as gastrointestinal, respiratory, and neurological. About 15% of CHIKF(+) patients presented with gastrointestinal signs and symptoms, including diarrhea, nausea, and gastroenteritis. A surprising 19.6% of CHIKF(+) patients presented with respiratory signs and symptoms. Most CHIKF(+) patients exhibiting gastrointestinal signs and symptoms were below the age of 15; patients exhibiting respiratory signs and symptoms were also largely under the age of 15 (Fig 3). No sign or symptom correlated to level of viremia, as determined by regression analysis of C_T_ bin against patient data (0.32<p<0.98; S3 Fig).

Given the high rate of hospitalization among CHIKF(+) patients relative to past outbreaks, the relative risk of presenting with specific signs and symptoms among admitted patients was assessed (Table C in S1 Tables). Firstly, while the mean age of admitted CHIKF(+) patients was lower than CHIKF(+) outpatients, this difference was not significant (ANOVA with Tukey’s post-hoc, p = 0.55); a similar observation was made for CHIKF(-) patients (ANOVA with Tukey’s post-hoc, p = 0.74). While fever and myalgia were not specifically associated with either admitted nor outpatient CHIKF(+) patients, admitted CHIKF(+) patients were 4.7 times more likely to present with arthralgia (95%CI = 1.03,21.06), 12.7 times more likely to present with headache (95%CI = 1.78, 90.18), 6.3 times more likely to present with dehydration (95%CI = 2.42, 16.67), 6.9 times more likely to present with gastrointestinal signs and symptoms (95%CI = 1.53, 30.84), and 9.6 times more likely to present with respiratory signs or symptoms (95%CI = 2.31, 40.05). Temperature was not significantly different between admitted and outpatient CHIKF(+) patients (ANOVA with Tukey’s post-hoc, p = 0.94), nor was time of symptom onset (ANOVA with Tukey’s post-hoc, p = 0.99). While no signs or symptoms imparted a significant risk of hospitalization among CHIKF(-) individuals, this finding may reflect the small number of patients in the CHIKF(-) group (n = 11 and n = 8 for admitted and outpatient groups, respectively) rather than the biological significance of respiratory and gastrointestinal features. Time of symptom onset was not significantly different between admitted and outpatient CHIKF(-) patients (p = 0.99).

**Fig 4:**
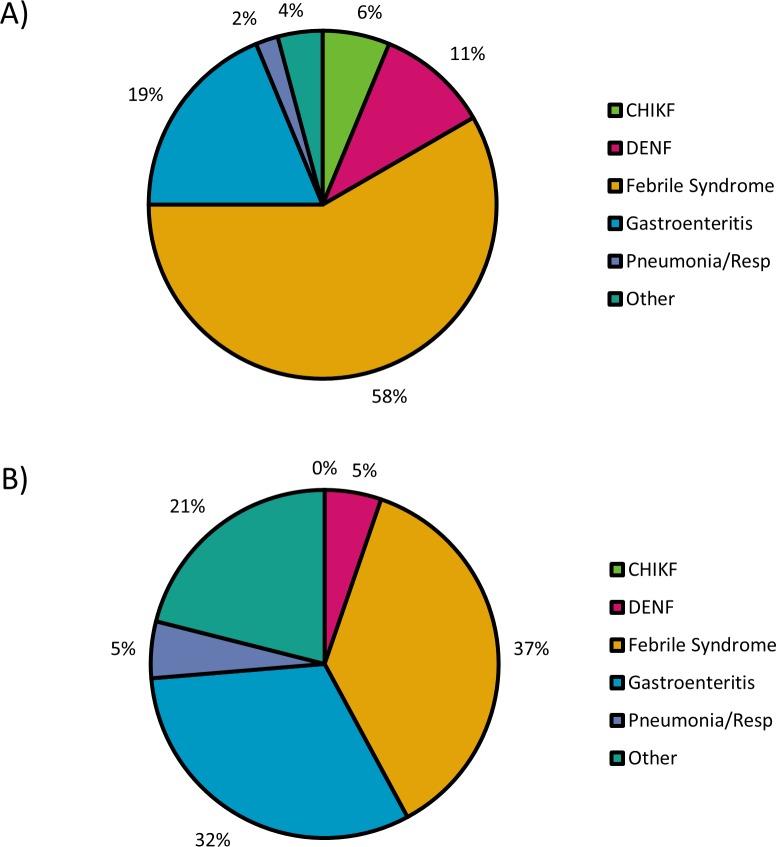
Clinical diagnoses made for CHIKF-positive and CHIKF-negative patients. Serum from patients visiting an emergency clinic in the DR was tested for CHIKV RNA and IgM, and results were retrospectively matched to initial diagnoses based on clinical presentation. The most common diagnosis for patients in both groups was undifferentiated febrile illness. Most notably, while CHIKF-positive (RNA-positive) patients (A) were more likely to be diagnosed with something other than CHIKF, no CHIKF-negative (RNA and IgM negative) patients (B) were misdiagnosed with CHIKF.

Initial diagnoses were made based on clinical findings (Fig 4). Only 6% of CHIKF(+) cases were clinically diagnosed as such, likely due to the absence of joint symptoms normally associated with CHIKF and possibly a lack of knowledge about CHIKF by some health care providers. CHIKF(+) patients were more likely to be diagnosed with idiopathic febrile syndrome, dengue fever, or pneumonia. No CHIKF(-) patients were misdiagnosed with CHIKF. Diagnoses classified under “other” included meningitis, pregnancy, asthma complications, and trauma. Some diagnoses for “generalized febrile syndrome” were inferred from the prescription of fever reducing agents, namely metamizole.

**Fig 5:**
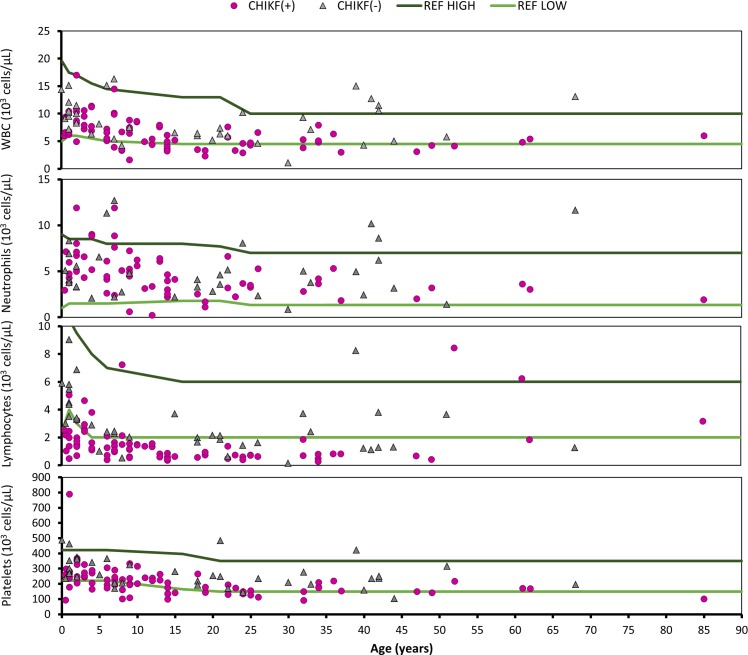
White blood cell values for CHIKF(+) and CHIKF(-) patients by age. Complete blood count (CBC) panel data for patients visiting an emergency clinic in La Romana, Dominican Republic, which were matched to Chikungunya virus (CHIKV) diagnostic results from discarded samples. Patients positive for CHIKV RNA by RT-qPCR are represented by solid purple circles, and patients negative for both CHIKV RNA and IgM antibodies [CHIKF(-)] are represented by grey triangles; green lines indicate suggested low and high reference values.

Pediatric (age<21) blood cell reference values were derived from standard hematology references [27], while adult reference values were provided by the laboratory at the Fundación Hospital General el Buen Samaritano. White blood cell (WBC) counts were generally unremarkable for both CHIKF(+) and CHIKF(-) groups, with the average for most age groups falling within normal ranges albeit with large variation; this also made statistical comparison of means impractical. Median values for complete WBC and lymphocytes were generally lower in CHIKF(+) patients than CHIKV(-) patients (Table 4). For CHIKF(+) patients: complete WBC ranged between 1.6–16.3x 10^3^ cells/μL; neutrophils ranged between 0.2–11.9x10^3^ cells/μL; lymphocytes ranged between 0.3–8.4x10^3^ cells/μL; and platelets ranged between 91-789x10^3^ cells/μL. CHIKF(-) patients showed similar ranges to CHIKF(+) patients: complete WBC ranged between 1.1–16.3x10^3^ cells/μL; neutrophils ranged between 0.8–12.7x10^3^ cells/μL; lymphocytes ranged between 0.2–9.0x10^3^ cells/μL; and platelets ranged between 103-487x10^3^ cells/μL. While most values fell within normal ranges for whole WBC, neutrophils, and platelets, many CHIKF(+) patients presented with varying degrees of lymphopenia when compared to reference values (Fig 5). Lymphopenia was significantly associated with relative level of viremia in CHIKF(+) patients, with the percent of patients presenting with lymphopenia decreasing with decreasing level of viral RNA; thrombocytopenia was not significantly associated with the level of viremia (linear regression with ANOVA, p = 0.006 and p = 0.081, respectively;S3 Fig), However, basal lymphocyte counts are highly specific to individuals, so it is possible that some of the deviant values fell within normal limits for some patients.

**Table 4:** Median values and interquartile range in parentheses for white blood cell (WBC) and differential counts for complete WBC, neutrophils, lymphocytes, and platelets by CHIKF positivity and age group.

Age	CHIKV+/-	WBC	Neutrophil	Lymphocyte	Platelets
**ALL**	**+**	6.2 (4.5–1.6)	4.2 (3.0, 6.1)	1.2 (0.7, 1.8)	205 (153.5, 249.5)
	**-**	8.2 (6.0, 11.2)	4.1 (3.0, 6.4)	2.4 (1.5, 3.7)	250 (205, 325)
**0–1**	**+**	6.6 (6.4,7.5)	4.28 (3.7, 5.4)	2.0 (1.3, 2.5)	265.5 (223.3, 286)
	**-**	10.2 (9.4, 12.7)	5.1 (4.1, 6.9)	4.5 (3.5, 5.8)	300 (274, 407)
**2–5**	**+**	9.1 (7.8, 10.8)	6.9 (5.5, 8.6)	1.9 (1.4, 2.9)	260.5 (244.5, 317.5)
	**-**	8.3 (8.1, 10)	3.3 (3.3, 5.6)	3.3 (2.9, 3.4)	341 (259, 364)
**6–14**	**+**	6.3 (4.3, 7.7)	4.6 (3.0, 6.1)	1.22 (0.8, 1.5)	213.5 (173.8, 238.5)
	**-**	7.5 (5.4, 15.1)	4.8 (2.7, 11.3)	2.3 (2.0, 2.4)	205 (205, 325)
**15–29**	**+**	4.5 (3.3, 5.3)	3.4 (2.3, 4.0)	0.7 (0.6, 0.7)	146.5 (132.3, 173.8)
	**-**	6.3 (6, 6.5)	3.6 (2.8, 4.6)	1.8 (1.6, 2.1)	234 (191, 253)
**30–49**	**+**	4.8 (3.8, 5.3)	3.2 (2.6, 3.8)	0.7 (0.5, 0.8)	152 (150, 179)
	**-**	9.3 (5, 11.5)	5.0 (3.2, 6.2)	1.3 (1.2, 3.7)	234 (198, 250)
**50–69**	**+**	4.8, (4.5, 5.1)	3.3 (3.2, 3.5)	6.24 (4.0, 7.3)	171 (169.5, 194)
	**-**	9.45 (7.6, 11.3)	6.5 (4.0, 9.1)	2.5 (1.9, 3.1)	255.5 (225.8, 285.3)

## Discussion

CHIKV infection was detected in 145 patients in La Romana, Dominican Republic by either RT-qPCR or IgM ELISA, and CHIKF-associated disease was described for patients; while only IgM antibodies were detected in 48 samples, CHIKV RNA was detected in 95 samples, providing the opportunity to isolate and sequence CHIKV. It is important to adequately characterize the molecular and clinical aspects of the CHIKV strain responsible for CHIKF outbreaks, which can cause financial harm to affected populations and severely affect quality of life. For example, it is estimated that the 2005–2006 CHIKF epidemic on La Réunion island cost €43.9 million (approx. $60.4 million USD at the time) for healthcare costs associated with infection [28]. This estimate excludes the cost of long-term treatment, typically for persistent arthralgia. Another study following a cohort of patients from the Réunion CHIKF epidemic from 2006 found that a significant portion of those infected with CHIKV continued to seek medical care up to 30 months after the acute infection resolved [29]. Similarly high costs are well documented through India, where medical expenses are out-of-pocket and thus present a significant financial challenge to affected families [30–32]. In Tolima, Colombia, 44.3% of patients with laboratory-confirmed CHIKV infection continued to suffer from polyarthralgia 7 months after their initial diagnosis, indicating that New World CHIKV infection may also result in chronic joint symptoms [33]. Although our study includes potential sources of bias, most importantly an interview bias inherent to the retrospective nature of the medical chart review, the results provide useful preliminary data for evaluating the clinical phenotype of Asian CHIKV genotypes circulating in the Americas.

Reports describing CHIKF outbreaks in the Caribbean and South and Central America caused by strains derived from the Asian lineage introduced in the Caribbean suggest that arthritis and arthralgia/joint pain are major symptoms. In Trinidad and Tobago, 83.3% of confirmed patients complained of joint pain, while arthralgia was reported in 96% of patients in Colombia [19, 34]. In our study, although arthritis was not reported by any physician, there was relatively little arthralgia associated with CHIKV infection, with only 20.0% of patients reporting joint pain. Furthermore, other symptoms heretofore considered typical of CHIKF—rash, headache, and myalgia—were nearly absent in La Romana. Colombia reported that 64% of CHIKF cases exhibited rash, 57% headache, and 24% myalgia, in contrast La Romana, where none of the patients evaluated exhibited rash, only 20% reported headache, and only 13.3% reported myalgia. These discrepancies may reflect sampling practices, as previous Caribbean outbreak studies have included only suspected cases of CHIKF and dengue while our study broadly evaluated all patients for whom a CBC test was ordered, regardless of clinical diagnosis. As such, cases of CHIKF were described in our study, which would not have otherwise been recognized as such without the characteristic joint symptoms and rash. In fact, many of the patients we studied visited the clinics for other indications ranging from gastrointestinal or respiratory infections to pregnancy-related issues, and CHIKF was only confirmed incidentally. Interestingly, in a prospective cohort study reported by Yoon et al, a CHIKF outbreak in the Philippines caused by a strain closely related to the Asian strains circulating in the Americas resulted in only 45% of patients reporting arthralgia [35]. The study by Yoon et al broadly evaluated febrile patients, supporting our symptomology findings in La Romana.

Historically, joint symptoms have been a hallmark of CHIKF. Outbreak descriptions from La Réunion and Italy, for example, found that 96.1% and 97% of laboratory-confirmed cases reported joint pain. Similarly, evaluation of an outbreak in Singapore caused by an Asian-lineage CHIKV strain found that 87.6% of patients reported arthralgia. Furthermore, rash, headache, and myalgia were also more prominent than in our study, with 38–52% of patients reporting skin rash, 40–51% reporting headache, and 46–60% reporting myalgia in Old World CHIKF outbreaks [15, 18, 36]. The reasons behind these discrepancies are unknown, but may include environmental factors such as diet, human genetic factors, as well as CHIKV lineage and strain-specific variation in pathogenesis. The diagnostic value of differential CBC analysis has been a focus of debate among CHIKV researchers, as some epidemics include a large majority of patients presenting with profound lymphopenia [15] while other studies show that changes in lymphocyte counts may not be CHIKV-specific compared to other indications, namely dengue hemorrhagic fever [37]. In La Romana, CHIKV infection greatly increased the risk of lymphopenia, given reference values derived from a combination of sources, when compared to CHIKF(-) patients. This suggests that, in La Romana, lymphopenia may potentially be used to distinguish CHIKF from other febrile illnesses. A more specific study comparing WBC results in dengue virus- and CHIKV-infected individuals would be needed to further substantiate this claim. However, in La Romana a larger percentage of children under the age of 14 were diagnosed as CHIKF(+) than previously reported. Over 62% of the cases we detected were children below the age of 14 and 50% were 10 or below. This is in stark contrast to past outbreaks, such as in La Réunion where only 5–14% of patients were 0–9 years of age and in Italy where only 6% of patients were 0–19 years old [15, 36, 38]. It is unclear whether this demographic difference reflects cultural differences, such financial constraints forcing the decision to treat a child instead an adult from a family with multiple febrile members, or a higher risk of CHIKV infection in children in La Romana due to differences in exposure to mosquitoes. As previously described, gastrointestinal CHIKF signs and symptoms such as nausea, vomiting, and diarrhea were more common among our younger patients. However, an overall larger percentage of patients presented with respiratory symptoms in La Romana compared to most CHIKF outbreaks. This increase in respiratory illness seen with CHIKF cases in the Americas is not restricted to La Romana—respiratory signs, namely cough, were also reported in 23% of cases in Trinidad [19]. Further, the CHIKF outbreak in the Philippines resulted in up to 40% of patients experiencing respiratory symptoms [35]. Although CHIKV is known to affect the cardiopulmonary system, causing heart palpitations, dyspnea, chest pain, and rarely death due to cardiac complications, it has not been shown to directly cause overt respiratory pathology [39–41]. Most likely, the respiratory illness associated with CHIKF in La Romana was either an exacerbation of existing conditions such as asthma, or co-infection with a respiratory pathogen. Respiratory co-infection in CHIKF cases confirmed by pathogen isolation has been documented in past outbreaks. In La Réunion Island during 2005–2006, Lemant and colleagues reported two patients over the age of 60 with laboratory-confirmed CHIKF who were initially diagnosed with pneumonia caused by *Streptococcus pneumoniae* and *Candida albicans*, respectively [39]. Additionally, in a sample of cases from a 2006–2007 outbreak in Pondicherry and Karaikal, India, 87% of laboratory-confirmed CHIKF patients were co-infected with respiratory syncytial virus (RSV) [42], and 9% were also infected with adenoviruses; just 4% of CHIKF cases were mono-infected with CHIKV. Curiously, only CHIKV(+) cases were co-infected with multiple respiratory viruses. In our study, five patients received a diagnosis of bronchopneumonia, two of pneumonia, and two of acute respiratory infection. However, it is unclear whether these diagnoses were confirmed with x-ray imaging. The relatively small number of CHIKF(+) patients with respiratory signs and symptoms, paired with the concurrently higher percentage of CHIKF(-) patients diagnosed with respiratory findings, suggests that CHIKV and respiratory co-infection is likely. Respiratory infections inflict a high burden of disease among Dominican children, and in 2002 accounted for 5% of all pediatric deaths [43]. Our retrospective chart review did not permit us to track the morbidity and mortality of patients following initial diagnosis and hospital admission. Nevertheless, respiratory failure and death in laboratory-confirmed CHIKF cases was a feature in the outbreaks in La Réunion, France, and in southern India [39, 42, 44, 45], where co-infection with respiratory pathogens was hypothesized to contribute to the elevated morbidity and mortality. Understanding how respiratory co-infections and underlying respiratory conditions may have contributed to morbidity and mortality in the 2014 Dominican Republic outbreak and in potential future outbreaks should be a research priority, and may be applicable to other areas in the Americas.

Other researchers have suggested that an attenuated disease phenotype may be associated with some Asian lineage CHIKV strains. Yoon et al. reported an unprecedented rate of subclinical CHIKV infections in the Philippines, with as many as 2–12 subclinical infections for every symptomatic infection, depending on the age group [35]. Similarly, Simmons et al. examined blood donation pools in Puerto Rico and found an alarming number of CHIKV RNA(+) samples, indicating that many pre-symptomatic or asymptomatic CHIKV-infected individuals donated blood [46]. Our study supports to these findings, indicating that typical markers of CHIKV infection, i.e. joint symptoms, were not as common among CHIKF(+) patients in La Romana. Further, we observed many more infections in children and adolescents, consistent with the finding that fewer asymptomatic cases were associated with younger patients in the study by Yoon *et a*l [35]. This may also explain the higher rate of respiratory signs and symptoms observed in La Romana and Trinidad, as children are naturally predisposed to respiratory infections. Genetically, CHIKV isolates from all of these outbreaks fall within the Asian lineage now circulating in the Americas [19, 35, 46]. While Zika virus RNA has been detected in the blood of a pregnant patient 10 weeks after symptom resolution [47], CHIKV RNA and/or antigen has only been shown to persist in joint and muscle tissue of humans (although no replicating virus has ever been isolated from individuals with chronic symptoms)[48, 49]. Therefore, rather than persistent viremia in CHIKV-infected patients biasing our symptom data, there could be an attenuation in the disease phenotype associated with American/Asian CHIKV strains compared to IOL strains.

Taken together, our data suggest that traditional symptom-based methods of diagnosing CHIKF may be insufficient in the Americas and other areas affected by recently emerging Asian lineage CHIKV strains; updating diagnostic approaches could greatly enhance surveillance in the Americas, an important consideration for designing vaccine and therapeutic clinical trials.

In summary, this study provided valuable clinical insights into the CHIKF outbreak in La Romana, DR with potential relevance to other areas in the New World where the Asian lineage of CHIKV is now circulating. Although limitations in our study design limit the ability to extrapolate these data to the Americas at large, the data the need to reevaluate current CHIKV surveillance practices and symptom-based diagnosis criteria.

### Limitations of our study

Our study succeeded in identifying unique clinical and laboratory factors present during the outbreak of CHIKF in the Dominican Republic in 2014. These data demonstrate new clinical characteristics that may be valuable for diagnostic and surveillance considerations in other areas affected by CHIKV strains closely related to the Asian strain introduced to the Caribbean. However, as an outbreak investigation, our study had several limitations including an inherent selection bias in that patients came from a single hospital in La Romana, which is not necessarily representative of a random sample of the Dominican population. The selection was further biased by the fact that RT-qPCR and IGM ELISA for CHIKV were run on all patients subjected to a CBC test. Reporting data, therefore, were sparse and inconsistent, and included patients who may or may not have experienced febrile symptoms. Since historical and physical exam data were not standardized, extracting them from clinical records created an information bias that included the possibility of various measurement errors (vital signs, interview information, etc.).

Finally, there appeared to be an information bias toward hospitalized patients, particularly in the CHIKV(-) group, as percent hospitalization increased with increasing level of information (i.e., symptoms were more likely to be recorded and/or records were more likely to be stored appropriately if a patient was admitted to the hospital). This is also a bias inherent to utilizing emergency clinic samples, as patients with severe illness will more likely visit the emergency clinic than those with minor (or no) symptoms, and thus a greater rate of hospital admissions will be detected in this particular patient population regardless of disease state. While this complicated our ability to interpret risk analyses when comparing CHIKF(+) to CHIKF(-) individuals, it did not necessarily affect our ability to detect symptoms in CHIKF(+) patients. Roughly 30% of the CHIKF(+) patients with symptom data were admitted to HBS; therefore roughly 30% of our population exhibited the most severe CHIKF symptoms (while 70% exhibited slight to moderate symptoms which did not necessitate hospital admission); even among the most severe CHIKF manifestations, only 30% of CHIKF(+) patients presented with arthralgia (compared to just 12% among the CHIKF(+) outpatient group). Furthermore, the failure to associate any sign or symptom (except lymphopenia) with the C_T_ value indicates that viremia was not an accurate predictor of disease severity. Along with the absence of fever in some patients with low to moderate C_T_ values, this suggests viremia in the absence of signs and symptoms as reported by Simmons *et al*, who found many blood donation pools to be strongly CHIKV RNA(+)[46]. Thus, information bias toward admitted patients and its complicating effect on comparing CHIKF(+) patients to the general CHIKF(-) population probably contributes to an overestimation of CHIKF symptoms among CHIKF(+) individuals.

## Supporting Information

S1 Fig C_T_ values generated from serum testing. Serum collected from discarded blood samples of patients visiting an emergency clinic in the DR was tested for CHIKV RNA. C_T_ values from positive results are displayed as bins (e.g., bin 17 represents 17.0≤C_T_<18.0)
(TIF)

S2 Fig Maximum clade credibility (MCC) phylogeny based on the complete coding region of 85 CHIKV sequences. Using an Illumina HiSeq platform, complete genome sequences were determined directly from RNA isolated from the sera of 10 individuals. The overall alignment rate of the reads varied widely among samples, ranging from 7–88%, with a mean of approximately 20%. Nucleotide and amino acid identity among the consensus sequences from these individuals was >99.9%. The MCC phylogeny showed that the Dominican Republic sequences clustered within the Asian lineage together with other Caribbean sequences isolated in 2014 [11, 16]. The most closely related Asian sequences circulated between 2012 and 2013 in Micronesia, the Philippines and China. The three major CHIKV genotypes are labeled. Nodes with clade credibility’s ≥ 95% are labeled accordingly.
(TIF)

S3 Fig C_T_ values generated from serum testing. Serum collected from discarded blood samples of patients visiting an emergency clinic in the DR was tested for CHIKV RNA and IgM, and samples testing positive for RNA were retroactively matched to clinical signs and symptoms documented by physicians (arthralgia, myalgia, enophthalmos, respiratory signs and symptoms, and gastrointestinal signs and symptoms) or results from complete blood count analysis (lymphopenia and thrombocytopenia). Results were organized by C_T_ bins defined by approximate log change from positive control (e.g., bin 20–22 represents samples within a 10-fold decrease from control, bin 23–25 within a 100-fold decrease, etc.). Only percent patients presenting with lymphopenia was significantly associated with C_T_ bin by linear regression analysis (p = 0.006), suggesting a positive correlation between percent patients presenting with lymphopenia and relative viremia.
(TIF)

S1 Tables Demographic and symptom comparisons between outpatient vs. admitted patients for both CHIKV-RNA positive and CHKV-negative serum samples. (DOCX)
