# Supplementary figures and images for: Molecular Virologic and Clinical Characteristics of a Chikungunya Fever Outbreak in La Romana, Dominican Republic, 2014
Source: PLoS Negl Trop Dis. 2016 Dec 28;10(12):e0005189. doi: 10.1371/journal.pntd.0005189 (PMC5193339; doi:10.1371/journal.pntd.0005189)

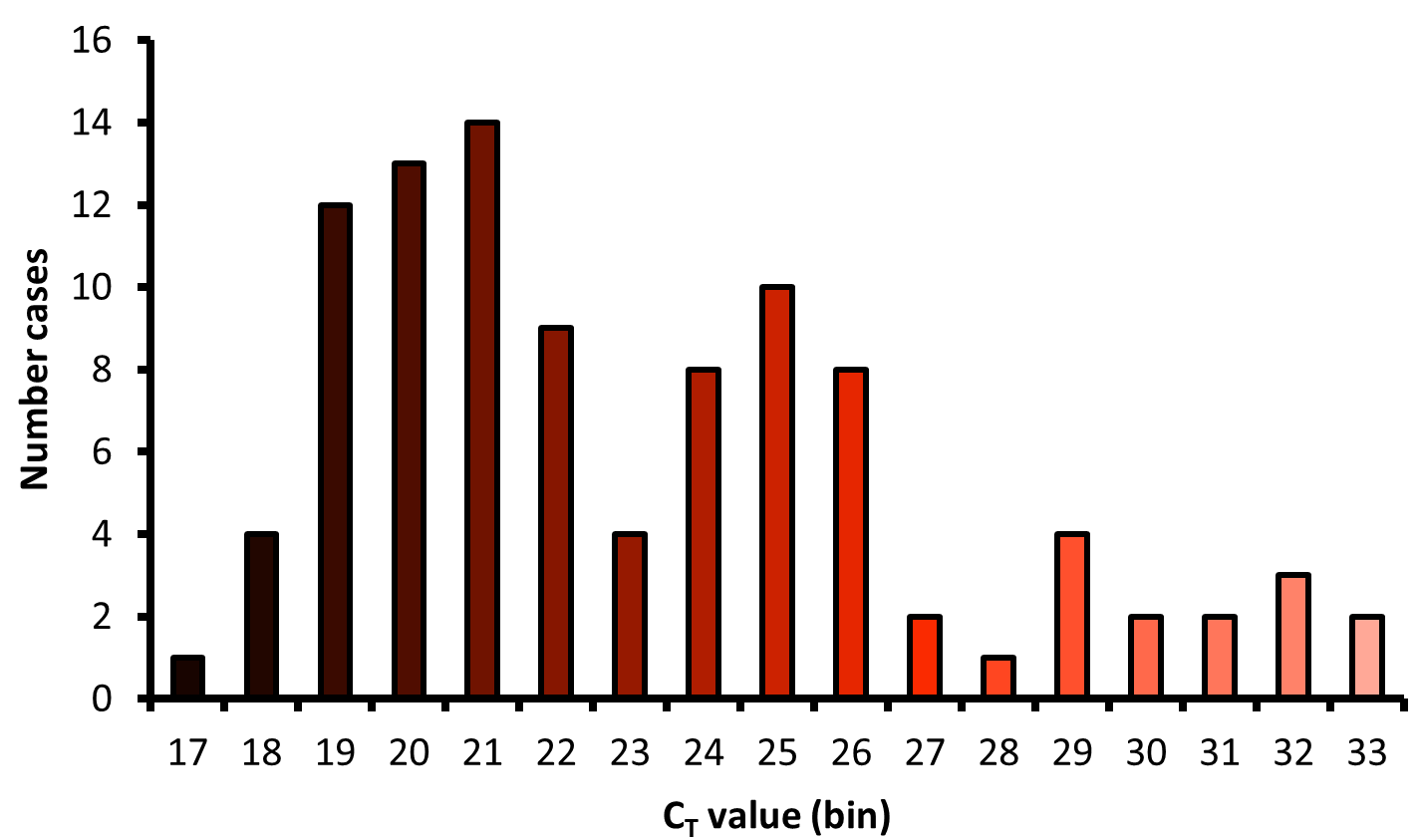

Supplement: S1 Fig — Serum collected from discarded blood samples of patients visiting an emergency clinic in the DR was tested for CHIKV RNA. CT values from positive results are displayed as bins (e.g., bin 17 represents 17.0≤CT<18.0) (TIF) [file pntd.0005189.s001.tif]

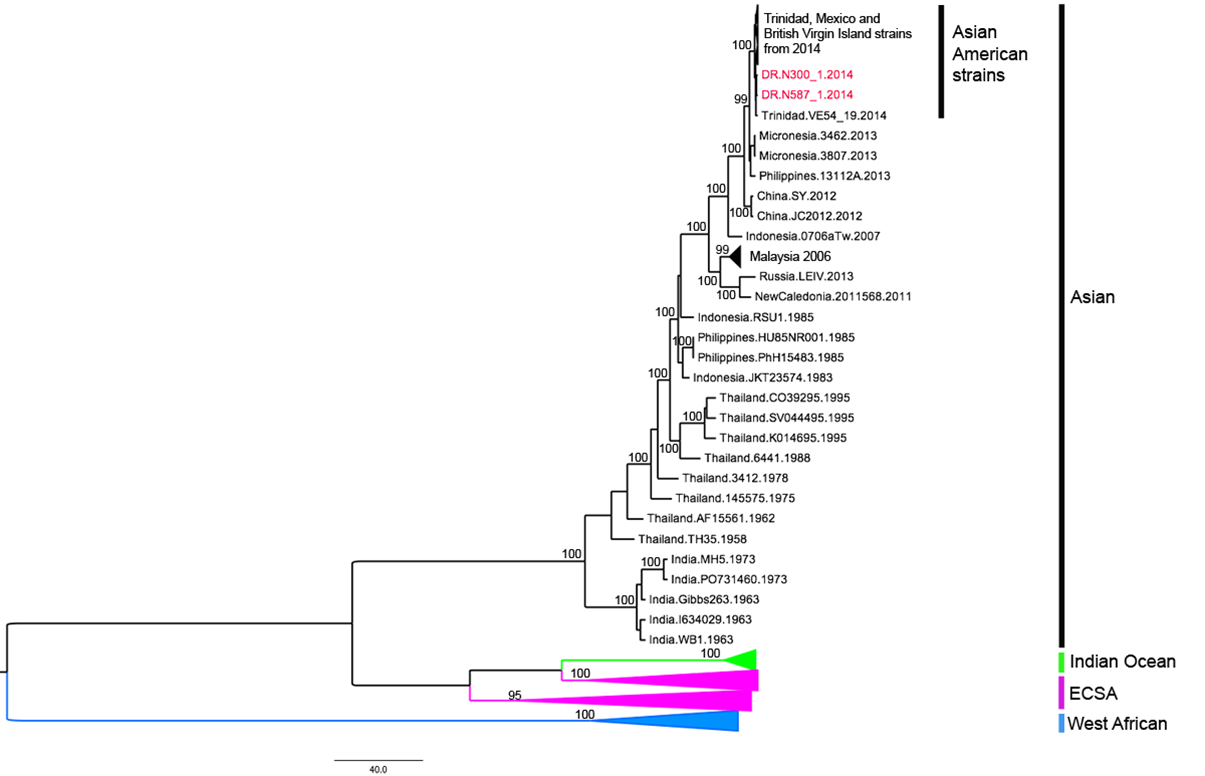

Supplement: S2 Fig — Using an Illumina HiSeq platform, complete genome sequences were determined directly from RNA isolated from the sera of 10 individuals. The overall alignment rate of the reads varied widely among samples, ranging from 7–88%, with a mean of approximately 20%. Nucleotide and amino acid identity among the consensus sequences from these individuals was >99.9%. The MCC phylogeny showed that the Dominican Republic sequences clustered within the Asian lineage together with other Caribbean sequences isolated in 2014 [11, 16]. The most closely related Asian sequences circulated between 2012 and 2013 in Micronesia, the Philippines and China. The three major CHIKV genotypes are labeled. Nodes with clade credibility’s ≥ 95% are labeled accordingly. (TIF) [file pntd.0005189.s002.tif]

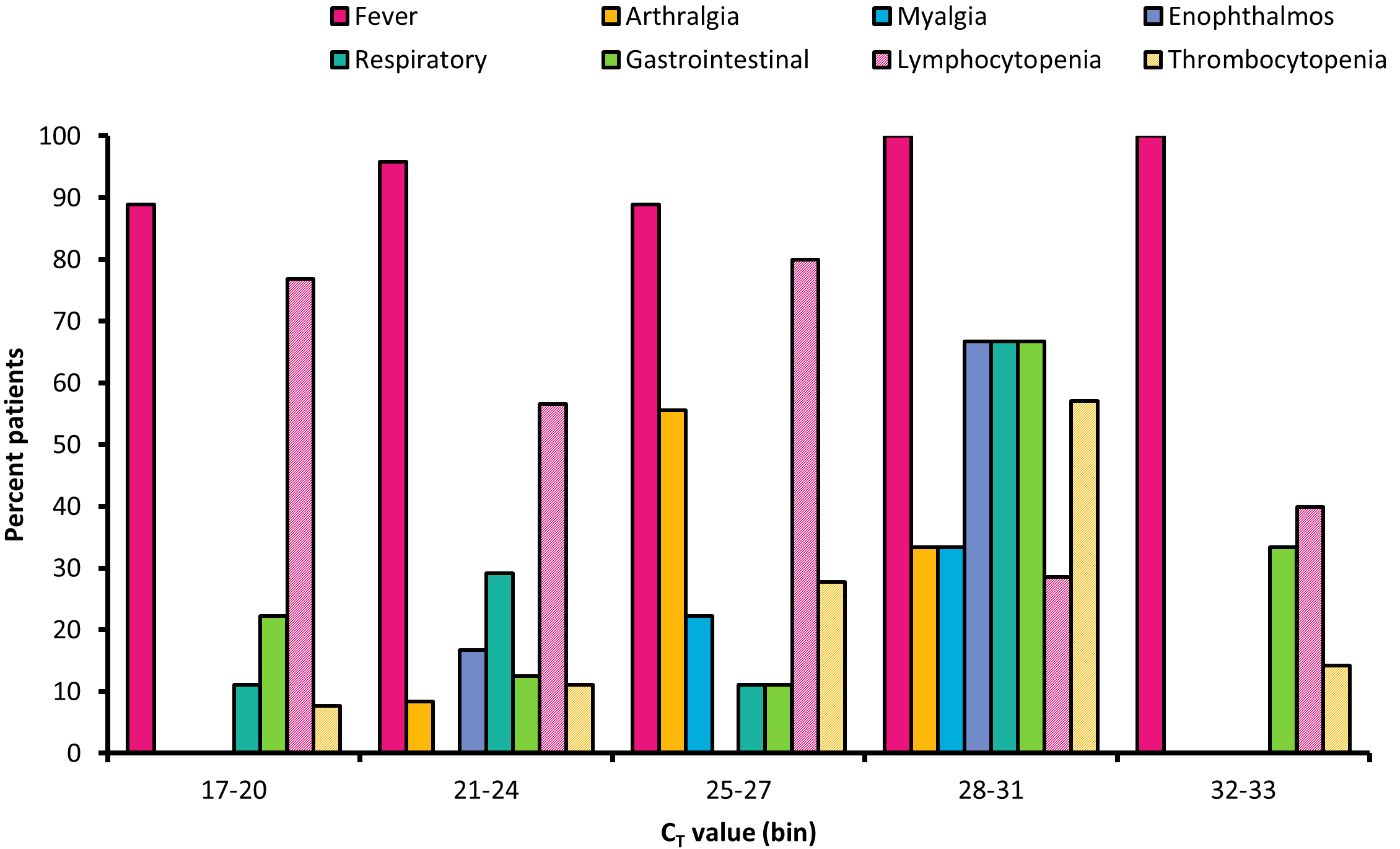

Supplement: S3 Fig — Serum collected from discarded blood samples of patients visiting an emergency clinic in the DR was tested for CHIKV RNA and IgM, and samples testing positive for RNA were retroactively matched to clinical signs and symptoms documented by physicians (arthralgia, myalgia, enophthalmos, respiratory signs and symptoms, and gastrointestinal signs and symptoms) or results from complete blood count analysis (lymphopenia and thrombocytopenia). Results were organized by CT bins defined by approximate log change from positive control (e.g., bin 20–22 represents samples within a 10-fold decrease from control, bin 23–25 within a 100-fold decrease, etc.). Only percent patients presenting with lymphopenia was significantly associated with CT bin by linear regression analysis (p = 0.006), suggesting a positive correlation between percent patients presenting with lymphopenia and relative viremia. (TIF) [file pntd.0005189.s003.tif]
