# Supplementary material for: Molecular Virologic and Clinical Characteristics of a Chikungunya Fever Outbreak in La Romana, Dominican Republic, 2014
Source: PLoS Negl Trop Dis. 2016 Dec 28;10(12):e0005189. doi: 10.1371/journal.pntd.0005189 (PMC5193339; doi:10.1371/journal.pntd.0005189)
Supplement: S1 Tables — (DOCX) [file pntd.0005189.s004.docx]

**Table A.** Comparison of outpatient (outpat) and admitted (admit) patient demographics for total patients with demographic information

|  | CHIKF(+) | | CHIKF(-) | |
| --- | --- | --- | --- | --- |
| **Characteristic** | Value outpat(admit) | Rel.Risk [95% CI]* | Value outpat(admit) | Rel.Risk [95% CI]* |
| **Male:Female (ratio)** | 1.74 (2.00) | 1.4 [0.8320, 2.3042] | 1.29 (1.67) | 1.0 [0.4591, 2.0075] |
| **Age (Average ±STDEV)** | 16.4±15.9  (10.5±9.1) |  | 36.4±17.56  (20.8±23.0) |  |
| **Time from onset to hospital visit, mean days ± STD** | 3.9±2.0 (3.7±1.7) |  | 4±1 (4±1.5) |  |

*Where applicable

**Table B.** Comparison of outpatient (outpat) and admitted (admit) patient demographics for symptomology data

|  | CHIKF(+) | | CHIKF(-) | |
| --- | --- | --- | --- | --- |
| **Characteristic** | Value outpat(admit) | Rel.Risk [95% CI]* | Value outpat(admit) | Rel.Risk [95% CI]* |
| **Male:Female (ratio)** | 0.93 (2.25) | 1.4 [0.8320, 2.3042] | 1.67 (1.5) | 1.0 [0.4591, 2.0075] |
| **Age (Average ±STDEV)** | 16.5±16.4 (9.8±9.6) |  | 29.6±23.3 (21.3±24.4) |  |
| **Time from onset to hospital visit, mean days ± STD** | 4.3±2.3 (3.7±1.7) |  | 3.5±0.7 (4.5±1.7) |  |

*Where applicable

**Table C.** Comparison of outpatient (outpat) and admitted (admit) patient symptomology

|  | **CHIKF(+)** | | **CHIKF(-)** | | |
| --- | --- | --- | --- | --- | --- |
| **Symptom** | **Outpat (admit)** | **Rel Risk [95%CI]** | | **Outpat (admit)** | **Rel Risk [95%CI]** |
| **Fever** | 90.9 (93.3) | 1.0 [0.8393, 1.2284] | | 50.0 (70.0) | 1.4 [0.6272, 3.1252] |
| **Average (°C ± STD)** | 39.4±0.7 (38.8±0.5) |  | | Not available (38.8±0.3) |  |
| **Arthralgia*** | 12.1 (33.3) | 4.7 [1.0339, 21.0641] | | 0 (0) | Incalculable |
| **Myalgia*** | 3.0 (13.3) | 6.3 [0.7604, 52.7477] | | 0 (0) | Incalculable |
| **Headache*** | 4.8 (66.7) | 12.7 [1.7791, 90.1827] | | 16.7 (66.7) | 4 [0.6125, 26.1239] |
| **Enophthalmos** | 0.0 (38.5) | 26.7 [1.5806, 451.5179] | | 0.0 (10.0) | 2.5 [0.1131, 53.2492] |
| **Malaise/fatigue** | 3.0 (23.0) | 7.6 [0.8692, 66.7179] | | 0.0 (20.0) | 4.1 [0.2238, 74.7824] |
| **Rash** | 0 (0) | Incalculable | | 0 (0) | Incalculable |
| **Dehydration** | 12.1(76.9) | 6.3462 [2.4160, 16.6695] | | 0.0 (50.0) | 11 [0.7401, 163.4975] |
| **Gastrointestinal^A^** | 6.1 (38.5) | 6.9 [1.5327, 30.8381] | | 12.5 (50.0) | 4 [0.5575, 27.7057] |
| **Respiratory^b^** | 6.1 (53.8) | 9.6 [2.3129, 40.0535] | | 25.0 (40.0) | 2 [0.5644, 7.0874] |

*Patient data for children under 3 years of age not included

^A^Nausea, diarrhea, vomiting ^B^pneumonia, dyspnea, ronchus, difficulty breathing, rhinorrhea

Values in percent unless otherwise noted.
